# Plasma and Liver Lipidomics Response to an Intervention of Rimonabant in ApoE*3Leiden.CETP Transgenic Mice

**DOI:** 10.1371/journal.pone.0019423

**Published:** 2011-05-17

**Authors:** Chunxiu Hu, Heng Wei, Anita M. van den Hoek, Mei Wang, Rob van der Heijden, Gerwin Spijksma, Theo H. Reijmers, Jildau Bouwman, Suzan Wopereis, Louis M. Havekes, Elwin Verheij, Thomas Hankemeier, Guowang Xu, Jan van der Greef

**Affiliations:** 1 CAS Key Laboratory of Separation Science for Analytical Chemistry, Dalian Institute of Chemical Physics, Chinese Academy of Sciences, Dalian, China; 2 Division of Analytical Biosciences, Leiden/Amsterdam Center for Drug Research, Leiden University, Leiden, The Netherlands; 3 Sino-Dutch Centre for Preventive and Personalized Medicine, Zeist, The Netherlands; 4 SU BioMedicine, Zeist, The Netherlands; 5 Gaubius Laboratory, TNO, Leiden, The Netherlands; 6 Department of Earth, Environmental and Life Science, TNO, Zeist, The Netherlands; 7 Netherlands Metabolomics Centre, Leiden/Amsterdam Center for Drug Research, Leiden University, Leiden, The Netherlands; Governmental Technical Research Centre of Finland, Finland

## Abstract

**Background:**

Lipids are known to play crucial roles in the development of life-style related risk factors such as obesity, dyslipoproteinemia, hypertension and diabetes. The first selective cannabinoid-1 receptor blocker rimonabant, an anorectic anti-obesity drug, was frequently used in conjunction with diet and exercise for patients with a body mass index greater than 30 kg/m^2^ with associated risk factors such as type II diabetes and dyslipidaemia in the past. Less is known about the impact of this drug on the regulation of lipid metabolism in plasma and liver in the early stage of obesity.

**Methodology/Principal Findings:**

We designed a four-week parallel controlled intervention on apolipoprotein E3 Leiden cholesteryl ester transfer protein (ApoE*3Leiden.CETP) transgenic mice with mild overweight and hypercholesterolemia. A liquid chromatography–linear ion trap-Fourier transform ion cyclotron resonance-mass spectrometric approach was employed to investigate plasma and liver lipid responses to the rimonabant intervention. Rimonabant was found to induce a significant body weight loss (9.4%, *p*<0.05) and a significant plasma total cholesterol reduction (24%, *p*<0.05). Six plasma and three liver lipids in ApoE*3Leiden.CETP transgenic mice were detected to most significantly respond to rimonabant treatment. Distinct lipid patterns between the mice were observed for both plasma and liver samples in rimonabant treatment vs. non-treated controls. This study successfully applied, for the first time, systems biology based lipidomics approaches to evaluate treatment effects of rimonabant in the early stage of obesity.

**Conclusion:**

The effects of rimonabant on lipid metabolism and body weight reduction in the early stage obesity were shown to be moderate in ApoE*3Leiden.CETP mice on high-fat diet.

## Introduction

Obesity, a major risk factor for serious diet-related chronic diseases such as diabetes and cardiovascular disease, is commonly stated as critically important compositions of the metabolic syndrome [1], [2]. In recent decades, obesity has reached epidemic proportions globally due to the rapid economic growth, modernization and urbanization. The major causes of its rising epidemic are excessive consumption of energy-dense food high in saturated fats and sugars and reduced physical activity [3], [4]. Obesity is known to be associated with dyslipoproteinemia characterized by increased levels of plasma triacylglycerides (TG) and low density lipoprotein cholesterol (LDL-C) and decreased level of high density lipoprotein cholesterol (HDL-C) [5]. Chronic liver disease associated with obesity has been identified in adults since 1970s and soon after this condition was also reported in childhood and adolescence [6], [7]. Most commonly, non-alcoholic fatty liver is observed in obese subjects with liver disease. This disease is frequently caused by complex hepatocellular metabolic dysfunctions due to the impaired insulin action, leading to disordered metabolism of fat and free fatty acids and subsequent oxidant mediated damage to the hepatocytes [6].

Traditionally, prevention and treatment of obesity focus on individual behavior interventions through increased regular exercise and a low-fat, low refined carbohydrate diet [4]. It has proven inadequate probably because the sociological factors are not taken into account. Medical treatment approaches for obesity have largely been developed in modern societies and appear to be effective on the short term [8], [9]. However, data reporting on long-term health outcome based on successful treatment strategies are very limited [10].

Previously, it has been demonstrated that early obesity is associated with endothelial dysfunction in high-fat fed pigs [11]. The observed abnormalities such as mild dyslipidemia, vascular oxidative stress and hypertension indicated that the early phases of obesity play a key role in the progression of coronary atherosclerosis and cardiovascular events and can be considered as the center point of the metabolic syndrome [6], [11]–[13]. Collectively, effective strategies for prevention and recognition of overweight and obesity in an early stage are critical.

Since lipids are involved in obesity-associated pathology, novel tools that enable a large-scale study of individual lipids in biological systems are highly demanded for understanding the potential pathogenic mechanisms. Lipidomics technology can provide an integrated view of lipid metabolites present in cells, tissues and biological fluids [14]–[16]. This tool can not only provide insights into the specific roles of lipids in monitoring health status, but will also assist in identifying potential preventive or biomarkers [17], [18]. The availability of novel analytical and advanced instrumental as well as powerful informatics technologies has facilitated the characterization of global changes of lipids in metabolic conditions such as obesity [19], insulin resistance [20], atherosclerosis [21], diabetes [22], and hepatic steatosis [23] and has facilitated data integration in order to understand the biological system.

Rimonabant, as the first selective cannabinoid-1 (CB_1_) receptor blocker, was proved to lead to reduced food intake, long-term maintained weight loss, and improved cardiovascular risk factors, manifesting as elevated plasma HDL-C, reduced plasma TG and inhibited insulin resistance in obese subjects [24]–[26]. In 2008 the European Medicines Agency withdrew the drug from the market in countries where it was commercially approved and marketed because of the psychiatric side-effects (e.g. depression and even suicide attempt) [27]. The aim of the current study was to unravel the underlying effects of rimonabant on plasma and hepatic lipid metabolism in the stage of early obesity.

For this we used a double transgenic mouse model, i.e. apolipoprotein E3 Leiden cholesteryl ester transfer protein (ApoE*3Leiden.CETP) transgenic mice, that matches with human lipid metabolism as closely as possible. The presence of ApoE*3Leiden hampers the uptake of very low density lipoprotein (VLDL)-remnants by the liver thus leading to increased VLDL/LDL-C levels in the plasma. CETP is a plasma glycoprotein that is responsible for the transportation of cholesterol ester (ChoE) from HDL to apoB-containing lipoproteins (e.g. VLDL and LDL) in exchange of TG, leading to decreased HDL-C levels [28], [29]. This gene is not present in wild type mice. Since in wild type mice the plasma cholesterol (Cho) is almost completely confined to the HDL fraction while VLDL and LDL are virtually absent due to the lack of CETP, wild type mice hardly develop dyslipidemia and, as a consequence, atherosclerosis [30]. The ApoE*3Leiden.CETP mice, however, have a higher VLDL/LDL-C level and relatively low HDL-C level. Taken together, ApoE*3Leiden.CETP mice have a human-like atherogenic lipoprotein profile. They not only respond in a human-like manner to pharmaceutical interventions with respect to lipid lowering efficacy [31], [32] but also respond to HDL modulating therapy. Many studies proved that the ApoE*3Leiden.CETP transgenic mouse is a valuable model to investigate the pathogenesis of vascular atherosclerotic lesion development and the effect of combination therapies on dyslipidemia and atherosclerosis [33]–[38]. In this paper we reported the results of the study of large-scale lipids in plasma and liver of 16 female ApoE*3Leiden.CETP mice, 8 of which were subjected to a period of 4 weeks of rimonabant intervention and 8 untreated animals.

Based on our study, we proposed that the rimonabant intervention on early obesity of ApoE*3Leiden.CETP mice would affect plasma and hepatic lipid metabolism relative to the non-treated controls, leading to increased HDL-C concentrations and decreased VLDL/LDL-C levels.

## Methods

### Ethics statement

All animals received humane care conforming to the rules and regulations set forward by the Netherlands Law on Animal Experiments. All animal experiments were approved by an independent institutional ethical committee on animal care and experimentation (Dierexperimenten Commissie DEC of Netherlands Organization for Applied Scientific Research, Zeist, the Netherlands) with a permit No. of DEC2489.

### Animals

ApoE*3Leiden.CETP transgenic mice, expressing a human CETP gene [34], were bred at TNO (Leiden, the Netherlands). In this study, sixteen female ApoE*3Leiden.CETP mice were used. All mice were housed under standard conditions in conventional cages (4 mice per cage) with free access to water and food. At the age of 6–10 weeks, mice were fed a semi-synthetic modified Western-type diet (Hope Farms, Woerden, Netherlands) containing 15% (w/w) saturated fat, 0.2% (w/w) Cho and 40% (w/w) sucrose as described by Nishina et al [39] as a 4 weeks run-in diet in order to get mildly elevated lipid levels (plasma Cho levels of about 14–18 mmol/L) and a moderate increase in body weight. Thereafter (t = week 0), mice were matched on body weight and plasma Cho and TG levels (after 4 h fasting) and separated into 2 groups. Subsequently, mice received a Western-type diet (Hope Farms, Woerden, Netherlands) without or with rimonabant (Sanofi-Aventis Netherlands B.V., Gouda, The Netherlands) at a concentration of 10 mg/kg body weight/day for a period of 4 weeks.

Table 1 presents the study design and time points at which both biochemical parameter and lipidomics profiling measurements were done.

**Table 1:** Study design and time points at which both biological parameters and lipidomic profiling were done.

Time points of experiment (week)	−4	−3	−2	−1	0	1	2	3	4
	Run-in period					Intervention			
						period			
Group 1, control	×	→			×	→			×
Group 2, rimonabant treatment	×	→			×	→			×
Randomization					×				
Body weight and food intake					×	×	×	×	×
Plasma cholesterol and triacylglyceride					×				×
Lipoprotein profile					×				×
HDL-C measurement					×				×
CETP level & activity					×				×
Sacrifice with plasma & liver collection for lipidomics									×

### Sacrifice and Sample Collection

Animals were sacrificed with rapid asphyxiation with CO_2_ and opened longitudinally after 4-week intervention experiment. Blood was collected before start of the intervention (t = week 0) and just before sacrifice (t = week 4) via tail vein bleeding into CB 300 LH microvettes (Sarstedt, Nümbrecht, Germany), containing lithium heparin and were placed on ice immediately after blood collection. Plasma samples were obtained after centrifugation of the blood samples for 10 min at 6000 rpm at 4°C. Liver tissues were dissected on ice and immediately weighted before being snap-frozen in liquid nitrogen. Both the plasma and the tissue samples were stored at −80°C until use.

### Plasma biochemical analyses and lipoprotein profile analysis

Plasma samples collected at t = week 0 and t = week 4 were assayed for total cholesterol (TC), total triacylglycerides (TG), HDL-C and lipoprotein profile. Plasma TC and TG were quantified using the commercially available enzymatic kits 236691 and 11488872 (Roche Molecular Biochemicals, Indianapolic, IN, USA), respectively. Plasma HDL-C was quantified after precipitation of apoB-containing lipoproteins. Pooled lipoprotein profiles were measured by fast performance liquid chromatography (FPLC) using an AKTA apparatus (Amersham Biosciences). Cho, TG and Phospholipid (PL) levels were measured in the fractions of freshly obtained samples. PLs were determined in the FPLC fractions using kit “phospholipids B” (Instruchemie Co., The Netherlands).

### Measurement of cholesteryl ester transfer activity in plasma

CETP level was measured in each animal using the enzymatic kit “RB-CETP” (Roar Biomedical, Inc.). The transfer of newly synthesized ChoE in plasma was assayed by a radioisotope method as described before [40]. Briefly, [^3^H] Cho mixed with bovine serine albumin was equilibrated with plasma free Cho for 24 h at 4°C followed by incubation for 3 h at 37°C. Subsequently, apoB-containing lipoproteins were precipitated by addition of heparin/MnCl_2_. Lipids were extracted from the precipitation and the labeled cholesteryl esters were separated from labeled unesterified Cho on silica columns and assayed by liquid scintillation counting.

### Lipidomics analyses

#### Lipid extraction for plasma samples

Briefly, 30 µl of internal standard (IS) mixture containing lyso-phosphocholine LPC (17∶0) at 1.5 µg/ml, phosphatidylethanolamine PE (34∶0) at 5 µg/ml, phosphatidylcholine PC (34∶0) at 5 µg/ml and TG (51∶0) at 5 µg/ml in 2∶1 of dichloromethane (CH_2_Cl_2_)/methanol (MeOH) and 30 µl of IS mixture containing LPC (19∶0) at 30 µg/ml, PE (30∶0) at 30 µg/ml, PC (38∶0) at 150 µg/ml and TG (45∶0) at 60 µg/ml in 2∶1 of CH_2_Cl_2_/MeOH were added to 30 µl of plasma which was placed in a new 2 ml eppendorf vial (Eppendorf, Hamburg, Germany). Following this, 180 µl MeOH and 360 µl CH_2_Cl_2_ were successively added. Thorough vortex was performed both before and after CH_2_Cl_2_ addition. Subsequently, 120 µl water was added to form a two-phase system in which lipids were located in the bottom organic phase. After 10 min centrifugation at a rotation speed of 6000 g at 10°C, 300 µl of lipid extracts from the bottom layer were transferred into a new brown auto-sampler vial. The extracts were diluted 20 times with acetonitrile/isopropanol/water (13∶6∶1, v/v/v) before LC–MS running.

#### Lipid extraction for liver samples

Sixty microliters of IS mixture containing LPC (17∶0) at 1.5 µg/ml, PE (34∶0) at 7.5 µg/ml, PC (34∶0) at 12.5 µg/ml and TG (51∶0) at 45 µg/ml in 2∶1 of CH_2_Cl_2_/MeOH and 60 µl of IS mixture containing LPC (19∶0) at 18 µg/ml, PE (30∶0) at 90 µg/ml, PC (38∶0) at 150 µg/ml and TG (45∶0) at 480 µg/ml in 2∶1 of CH_2_Cl_2_/MeOH were added to 10 mg of dry liver powder followed by addition of 160 µl of MeOH containing 0.02% antioxidant butylated hydroxytoluene, and then 320 µl of CH_2_Cl_2_ was added. The mixture was thoroughly vortexed for 1 min both before and after CH_2_Cl_2_ addition. After that, the resulted suspension was placed for 5 min in an ultrasonic bath at −4°C and then placed in a shaker followed by 45 min incessantly shaking at −4°C. A 10 min centrifugation at a rotation speed of 6000 g at 10°C was needed before 500 µl of the supernatant was transferred into a new 2 ml eppendorf vial. Subsequently, 100 µl of 0.9% NaCl was added to the supernatant to give rise to a two-phase system: the nonlipid compounds were located in the upper aqueous phase, while most of the lipids were in the lower organic phase. After being centrifuged at 2000 g for 10 min at 10°C, a total of 300 µl of lipid extract was collected from the bottom organic phase. Diluted the lipid extracts 40× with acetonitrile/isopropanol/water (13∶6∶1, v/v/v); 10 µl was loaded for LC–MS lipidomics analysis.

#### LC–MS lipid profiling

Diluted lipid extracts from both plasma and liver tissue samples were measured on a liquid chromatography–linear ion trap-Fourier transform ion cyclotron resonance-mass spectrometric (LC–FTMS) system equipped with a Surveyor HPLC MS pump, an autosampler (Thermo Fischer, San Jose, CA) and an Ascentis Express C8 2.1×150 mm (2.7 µm particle size) column (Sigma-Aldrich Chemie B.V., Zwijndrecht, The Netherlands). The binary solvent consisted of water/acetonitrile (2∶3, 10 mM ammonium formate) and acetonitrile/isopropanol (1∶9, 10 mM ammonium formate). The LC separation conditions were identical to those described previously [41]. The lipidomics profiling was carried out in the full ESI positive ion mode with a mass range of m/z 430–1500. The heated capillary was set at 300°C. The voltages of the sampling cone and capillary were 3.8 kV and 48 V, respectively. The tube lens was optimized to be 140 V. Nitrogen was used as sheath gas (60 units), auxiliary gas (5 units) and sweep gas (3 units). The LC–MS data were acquired by Xcalibur (Thermo Fisher) with 200 ms maximum injection time. The number of μscans was 2. Both the ion trap and FT scan events were recorded during data acquisition.

Specifically, samples of interest (i.e. plasma or liver samples) were randomly analyzed and the quality control (QC) samples, prepared by pooling of all plasma or liver samples, were regularly placed in the measurement sequence. Of note, plasma and liver samples were analyzed separately. General information of 8 exogenous lipid standards used for lipidomics analyses were summarized in the Supporting Information Table S1. The spiked concentrations of 8 exogenous lipid standards used in lipidomics analyses and normalization strategies used for LC–MS lipidomics data analyses were summarized in the Supporting Information Table S2.

#### Preprocessing of lipidomics data

Lipid peaks including spiked IS such as LPC (17∶0), LPC (19∶0), PE (34∶0), PE (30∶0), PC (34∶0), PC (38∶0), TG (51∶0) and TG (45∶0) were extracted based on their expected retention time and accurate masses according to an in-house lipid database using LCquan v2.5 (Thermo Fisher). The peak area of each extracted lipid ion was calibrated by an appropriate IS. Duplicate measurements were averaged to a single measurement after IS calibration. Data quality was assessed by calculating the relative standard deviation (RSD) of all calibrated lipid peaks in the QC samples. Peaks with a RSD larger than 20% were excluded leaving 131 lipids in the plasma lipidomics data set and 133 lipids in the liver lipidomics data set for subsequent data analyses (see the Supporting Information Table S3).

General lipidomics protocol and lipidomics platform characteristics such as linearity, repeatability and recovery were provided in the Supporting Information Text S1, Figures S1, S2, S3 and Tables S4, S5, S6, S7, S8.

### Statistical analysis

Statistical significance of biochemical parameters was analyzed by independent student t-test. Lipidomics data were first analyzed by independent student t-test and later extended with Benjamini and Hochberg multiple testing corrections. Data were expressed as mean ± SD unless otherwise stated. A value of *p*<0.05 was considered statistically significant.

In order to visualize possible relations between the samples from treated and non-treated groups, principal component analysis (PCA) was carried out for the mean centered plus unit variance scaled plasma lipidomics data and liver lipidomics data, respectively using Matlab software (version 6.5.1, release 13, The Mathworks, 2003).

One control mouse (marked as 3733) was excluded from statistical data analyses, because it did not respond to Western-type diet during run-in period and failed to reach hypercholesterolemia criteria essential for our experiment. We observed that the relative levels of most hepatic lipids were much lower in this mouse as compared to the other control mice. In this animal, the biochemical markers such as plasma TC, TG and liver weight were lowest among all control mice (data not shown).

## Results

### Food intake and body weight

**Figure 1:**
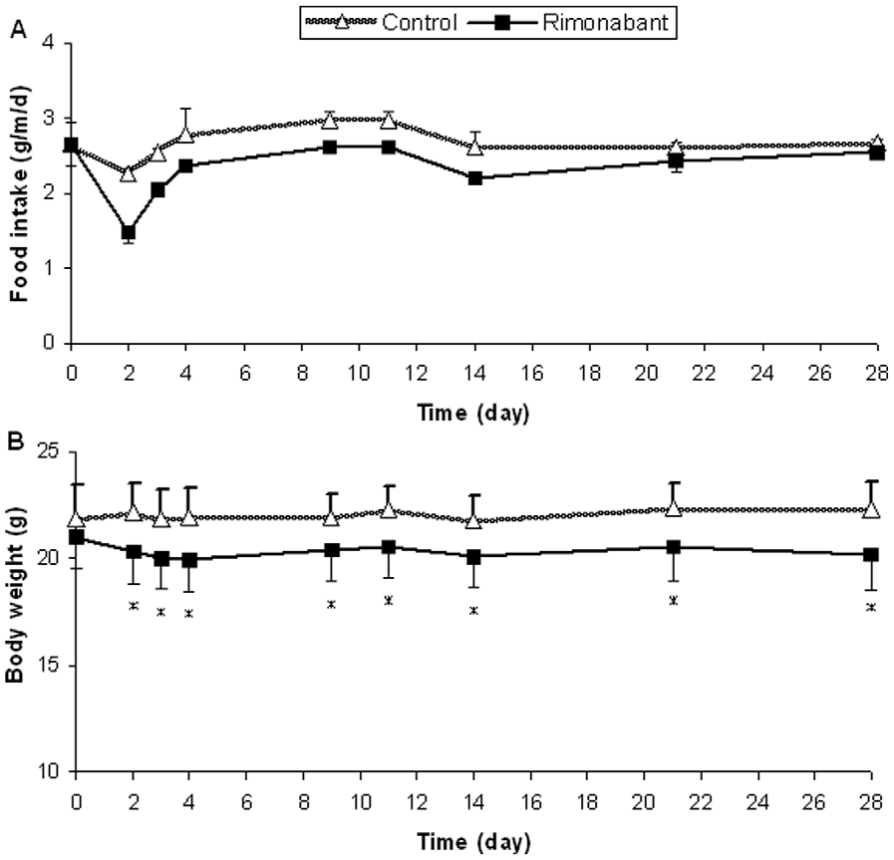
Food intake and body weight. The 4-week intervention effect of rimonabant on food intake and body weight was evaluated by measuring body weight (per mouse) and food intake (per cage) of the rimonbant and nontreated control mice at day 0, 2, 3, 4, 9, 11, 14, 21 and 28 respectively. (A) Food intake. (B) Body weight. Values are expressed as means ± SD; * *p*<0.05 vs. the control.

The variation in food intake and body weight during the 4 weeks of intervention is shown in Figure 1A and B, respectively. The body weight was significantly reduced in mice on rimonabant compared to control throughout the whole intervention period. In total, the weight loss was 9.4% (*p* = 0.03) at the end of the experiment. This decline in body weight might be explained by reduced food intake in the initial states of the experiment, although statistical significance was not reached.

### Plasma cholesterol, triacylglycerides, HDL-C and plasma lipoprotein profiles

**Figure 2:**
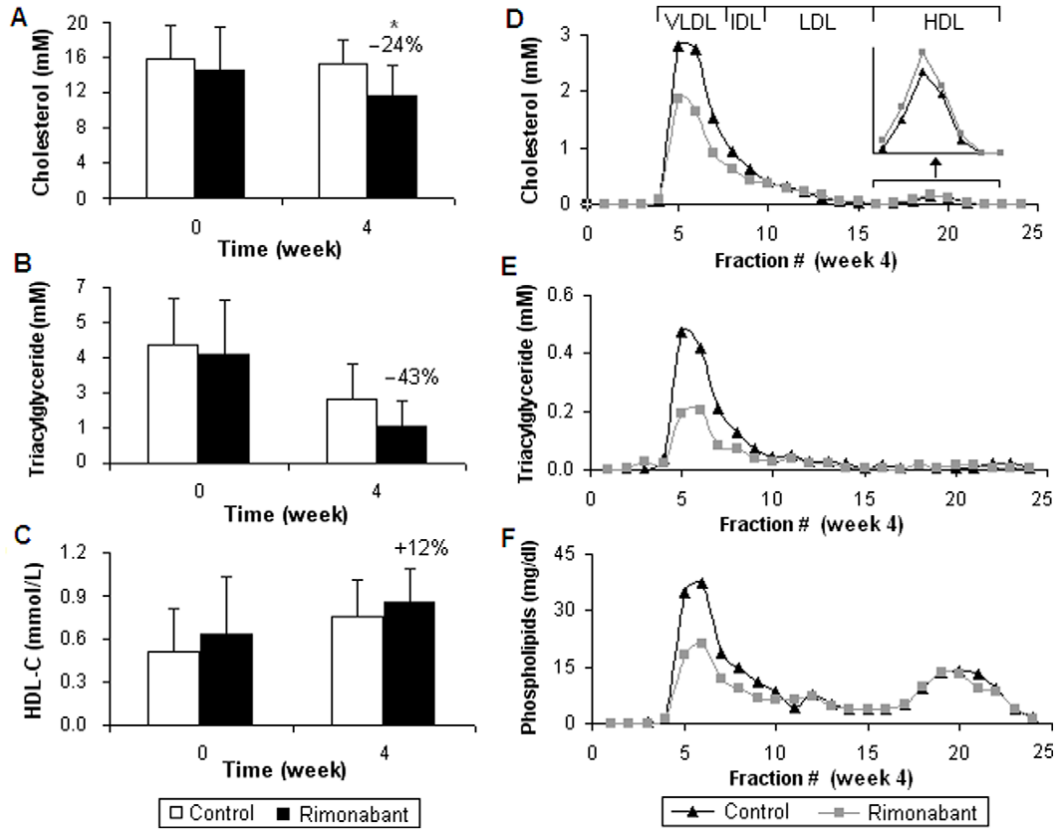
Plasma cholesterol, triacylglycerides, HDL-C and plasma lipoprotein profiles. Plasma concentrations are shown for TC (A), TG (B) and HDL-C (C) of the rimonabant and nontreated mice at week 0 and 4. Values are shown as means ± SD; * *p*<0.05 vs. the control. Alterations of Cho (D) and TG (E) as well as PLs (F) in the pooled lipoprotein profiles of the rimonabant treatment group and the controls (t = week 4). Fractions 4–7 represent VLDL; fractions 8–9 represent intermediate-density lipoprotein (IDL); fractions 10–15 represent LDL; fractions 16–23 represent HDL.

After a period of 4 weeks of intervention, plasma TC was significantly reduced by 24% (*p* = 0.04) (Figure 2A) and plasma TG reached a reduction trend (e.g. 1.34±0.96 vs. 2.35±1.34 mM, *p* = 0.11) in the rimonabant group as compared to the control mice (Figure 2B). As compared to the control, we could not see a significant increase in plasma HDL-C upon rimonabant intervention (Figure 2C).

The 4-week rimonabant intervention led to decreased levels of Cho, TG and PLs in the VLDL for 1.5, 2.5 and 2 fold respectively (Figure 2D–F) and to a slightly increased level of Cho in HDL particles (magnified part in Figure 2D). Concentrations for Cho and TG as well as PLs were unaffected in LDL particles, whereas TG and PL concentrations were unaffected in HDL particles.

### Rimonabant does not significantly affect plasma CETP activity

**Figure 3:**
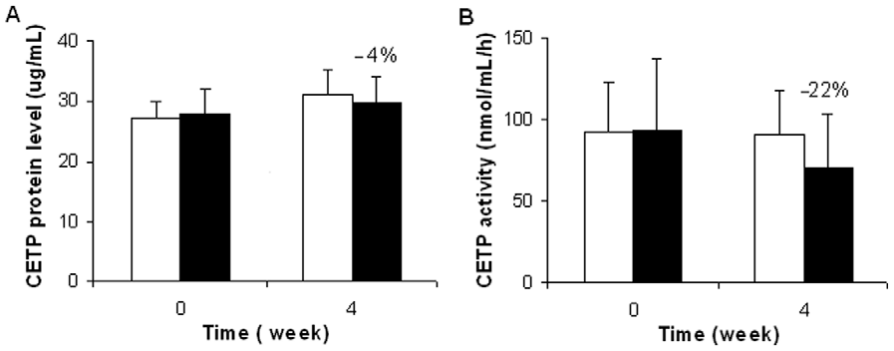
Rimonabant does not significantly affect plasma CETP activity. Effect of rimonabant on plasma CETP was evaluated by CETP protein level (A) and CETP activity (B) in ApoE*3Leiden.CETP mice at t = week 0 and 4 (white bars: the control group; black bars: the rimonabant group). Values are means ± SD. There were no statistically significant changes found in either CETP or CETP activity during the intervention treatment.

The CETP level was constant during the intervention (Figure 3A). The four-week rimonabant intervention resulted in a non-significant change of plasma CETP activity (e.g. 90.8±27.0 vs. 70.6±33.3 nmol/ml/h, *p* = 0.22) as compared to the control (Figure 3B).

### Lipidomics reveals differences between non-treated and rimonabant-treated mice for both plasma and liver samples

**Figure 4:**
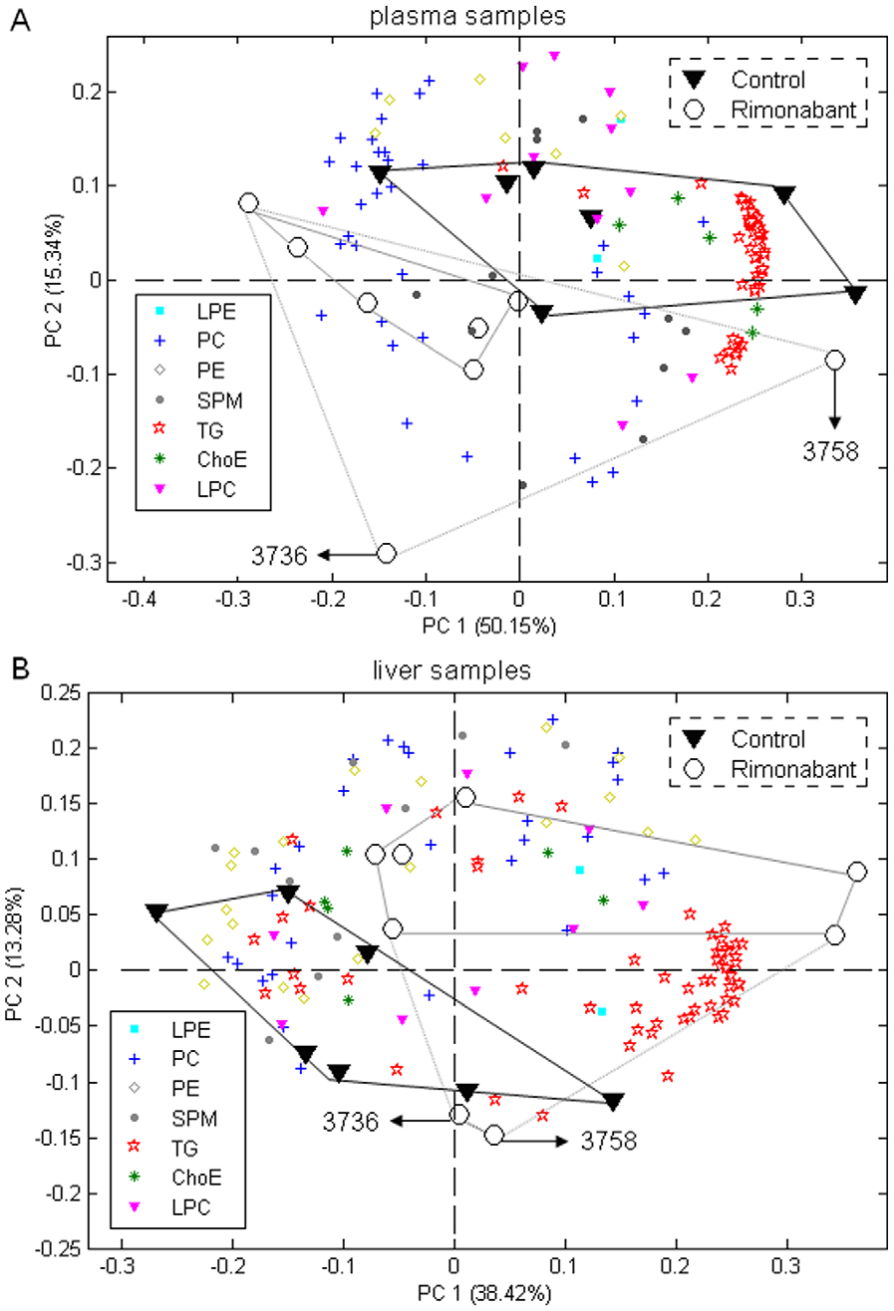
Lipidomics reveals differences between non-treated and rimonabant-treated mice for both plasma and liver samples. Principle component analyses (PCA) of plasma and liver lipidomics data were applied to differentiate the nontreated controls (n = 7) and the animals treated with rimonabant (n = 8) in plasma and liver samples, respectively. PCA biplots for the first two principal components of lipid profiling of plasma samples (A) and liver samples (B). Both PCA models used mean centred plus unit variance scaled data.

To get an overview of existing patterns in the lipidomics data such as clusters of mice of nontreated controls and mice undergoing rimonabant treatment and which lipids contributed most to these clusters, we performed PCA for the plasma and liver lipidomics datasets, respectively. Figure 4 displays the PCA biplots (A, plasma samples; B, liver samples). In both plasma and liver the rimonabant treated group was separated well from the control group. Two rimonabant treated mice (marked as 3736 and 3758) deviated from the others within the group causing some overlap with the mice from the control liver group. The deviations of these two mice from other group members were further checked with data from biological parameters and lipidomics. The biological parameters revealed that among all rimonabant treated mice the liver weights of these two mice were the heaviest and the total plasma TG levels were the lowest (data not shown). The liver lipidomics data showed that the TG levels were more abundant in these two mice than other rimonabant treated mice. In addition, the loadings in the biplot (lipid species represented by colored symbols in Figure 4A and B) indicated that TG lipid species dominated the differentiation between non-treated controls and the rimonabant-treated mice for both plasma and liver lipidomics data sets.

### Rimonabant significantly affects individual lipids in both plasma and liver of treated mice

In order to investigate quantitative changes of lipids in the rimonabant intervention group as compared to the control, statistical significance for the mean difference of all identified lipids between the rimonabant group and the control group was tested using independent student t-test. It was found that 33 plasma and 25 liver lipid species out of 131 and 133 lipids respectively were significantly changed after the 4-week rimonabant intervention as compared to the controls. Four lipids changed significantly upon rimonabant treatment in both plasma and liver, i.e. PE (36∶3), TG (50∶1), TG (52∶2) and TG (56∶7). Within these perturbed lipids, one interesting observation was that 31 out of 33 lipid species were significantly *decreased* in plasma whereas 22 out of 25 lipid species were significantly *increased* in liver in the rimonabant group versus the controls (Table 2 and 3).

**Table 2:** Lipid molecular species significantly changed in plasma of ApoE*3Leiden.CETP mice upon the rimonabant treatment as compared to the untreated controls.

			Rimonabant		
Lipid species	Control	Rimonabant	vs. control		Up (↑) or
	(mean ± SD)	(mean ± SD)	change (%)	*p* value	down (↓)
LPC (18∶1)	0.2896±0.0146	0.2414±0.032	83	**0.0030** **	↓
LPC (18∶2)	0.1402±0.0105	0.1069±0.0138	76	**0.0002** ***	↓
LPC (20∶3)	0.0264±0.0023	0.0219±0.0034	83	0.0109*	↓
LPE (20∶4)	0.0022±0.0001	0.0017±0.0002	76	**0.0001** ***	↓
PC (36∶2)	0.0848±0.0088	0.0738±0.0059	87	0.0133*	↓
PC (38∶2)	0.5067±0.0589	0.3773±0.0624	74	**0.0012** **	↓
PE (36∶3)	0.7573±0.1050	0.6087±0.1137	80	0.0214*	↓
PE (38∶2)	0.2678±0.0457	0.2042±0.0205	76	**0.0035** **	↓
PE (38∶4)	0.1453±0.0243	0.1126±0.0146	77	0.0068**	↓
SPM (16∶0)	0.0046±0.0003	0.0054±0.0004	118	**0.0006** ***	↑
SPM (18∶0)	0.0008±0.0001	0.0010±0.0001	122	0.0102*	↑
ChoE (18∶1)	0.1187±0.0171	0.0917±0.0210	77	0.0180*	↓
TG (44∶0)	0.0169±0.0009	0.0151±0.002	89	0.0437*	↓
TG (46∶0)	0.0328±0.0037	0.0273±0.0047	83	0.0259*	↓
TG (46∶1)	0.0127±0.0038	0.0078±0.0033	62	0.0198*	↓
TG (48∶0)	0.0167±0.0057	0.009±0.0056	54	0.0202*	↓
TG (48∶1)	0.0084±0.0028	0.0048±0.0032	57	0.0387*	↓
TG (48∶2)	0.1037±0.0302	0.0558±0.0411	54	0.0247*	↓
TG (50∶0)	0.0493±0.0196	0.0251±0.0197	51	0.0329*	↓
TG (50∶1)	0.1568±0.0685	0.0805±0.0631	51	0.0429*	↓
TG (52∶2)	1.0304±0.4185	0.5248±0.4136	51	0.0353*	↓
TG (52∶6)	0.0025±0.0008	0.0013±0.001	52	0.0298*	↓
TG (54∶2)	0.3189±0.1698	0.1508±0.1247	47	0.0459*	↓
TG (54∶3)	0.9160±0.3800	0.4568±0.3700	50	0.0341*	↓
TG (54∶4)	0.1780±0.0750	0.0921±0.0693	52	0.0382*	↓
TG (56∶3)	0.1001±0.0481	0.0453±0.0314	45	0.0200*	↓
TG (56∶4)	0.0765±0.0328	0.0396±0.0253	52	0.0289*	↓
TG (56∶7)	0.3749±0.1634	0.2055±0.134	55	0.0459*	↓
TG (58∶3)	0.0057±0.0028	0.0029±0.0017	52	0.0367*	↓
TG (58∶4)	0.0051±0.0022	0.0028±0.0014	55	0.0290*	↓
TG (58∶5)	0.0155±0.0069	0.0084±0.005	54	0.0384*	↓
TG (58∶6)	0.0162±0.0055	0.0098±0.0044	60	0.0253*	↓
TG (58∶7)	0.0264±0.0091	0.016±0.0078	60	0.0322*	↓

*P<0.05,

**p<0.01,

***p<0.001 vs. the untreated controls.

*P* values correspond to the mean difference between the rimonabant treatment and the untreated controls.

*Note*: lipids with *p* values marked in bold mean those remain significant after multiple testing correction.

**Table 3:** Lipid molecular species significantly changed in liver of ApoE*3Leiden.CETP mice upon the rimonabant treatment as compared to the untreated controls.

			Rimonabant		
Lipid species	Control	Rimonabant	vs. control		Up (↑) or
	(mean ± SD)	(mean ± SD)	change (%)	*p* value	down (↓)
PC (38∶4)	2.160±0.299	2.563±0.319	119	0.0262*	↑
PC (40∶4)	0.157±0.012	0.196±0.024	125	**0.0018** **	↑
PC (40∶5)	0.834±0.069	1.052±0.184	126	0.0113*	↑
PC (40∶6)	0.546±0.048	0.668±0.086	122	0.0054**	↑
PC (40∶8)	0.272±0.040	0.225±0.036	83	0.0318*	↓
PE (34∶1)	0.254±0.038	0.308±0.055	121	0.0484*	↑
PE (36∶3)	0.529±0.126	0.418±0.066	79	0.0493*	↓
PE (38∶6)	1.474±0.079	1.785±0.341	121	0.0355*	↑
PE (40∶6)	0.563±0.057	0.711±0.125	126	0.0133*	↑
ChoE (16∶1)	0.628±0.232	1.078±0.160	172	**0.0007** ***	↑
ChoE (22∶6)	0.043±0.008	0.054±0.005	127	0.0054**	↑
TG (50∶1)	1.357±0.363	1.773±0.362	131	0.0450*	↑
TG (50∶2)	1.779±0.570	2.497±0.636	140	0.0396*	↑
TG (50∶3)	0.347±0.132	0.581±0.211	167	0.0251*	↑
TG (52∶2)	7.234±1.170	8.572±1.160	118	0.0448*	↑
TG (52∶4)	0.462±0.116	0.679±0.229	147	0.0411*	↑
TG (52∶5)	0.526±0.165	0.840±0.346	160	0.0478*	↑
TG (54∶5)	0.354±0.078	0.504±0.136	143	0.0235*	↑
TG (54∶6)	0.616±0.181	1.021±0.305	166	0.0091**	↑
TG (54∶7)	0.078±0.025	0.141±0.058	180	0.0195*	↑
TG (56∶5)	0.517±0.100	0.687±0.151	133	0.0250*	↑
TG (56∶6)	0.135±0.027	0.209±0.033	155	**0.0005** ***	↑
TG (56∶7)	1.047±0.281	1.562±0.558	149	0.0461*	↑
TG (56∶8)	0.168±0.049	0.274±0.108	163	0.0326*	↑
TG (60∶1)	0.015±0.002	0.012±0.002	83	0.0352*	↓

*P<0.05,

**p<0.01,

***p<0.001 vs. the untreated controls.

*P* values correspond to the mean difference between the rimonabant treatment and the untreated controls.

*Note*: lipids with *p* values marked in bold mean those remain significant after multiple testing correction.

Remarkably, after multiple testing correction only 6 plasma lipids (LPC-18∶1, LPC-18∶2, LPE-20∶4, PC-38∶2, PE-38∶2 and sphingomyelin SPM-16∶0) and 3 liver lipids (PC-40∶4, ChoE-16∶1 and TG-56∶6) remained significant out of 33 and 25 plasma and liver lipids respectively (lipids with *p* values marked in bold in Table 2 and 3).

### Rimonabant significantly decreases the overall responses of plasma lipid classes

**Figure 5:**
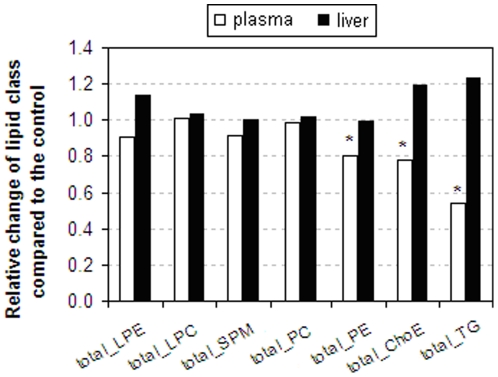
Rimonabant significantly decreases the overall responses of plasma lipid classes. Relative change of the summation of the individually measured lipids in different lipid classes was determined based on plasma and liver lipidomics data of the rimonabant group and the controls (white bars: plasma samples; black bars: liver samples). The summation of the individually measured lipids in each lipid class in the control is set to be 1, * *p*<0.05 vs. the control.

The summation of the individually measured lipids into different lipid classes in plasma showed a significant reduction of PE with 20% (*p* = 0.02), ChoE with 22% (*p* = 0.02) and TG with 46% (*p* = 0.04) in rimonabant treated mice vs. the control (white bar graph in Figure 5); the summation of individually measured lipids into lipid classes of LPC, PC and SPM in plasma of mice receiving rimonabant treatment were comparable to those in untreated controls under the current LC–MS conditions. In total, the level of PLs in plasma samples was comparable in rimonabant treated mice vs. the control (data not shown). In liver tissues, none of the summation of the individually measured lipids within each of lipid classes was significantly changed after the rimonabant intervention in relation to the control (black bar graph in Figure 5).

## Discussion

Rimonabant, a selective CB_1_ receptor antagonist, is known for reducing body weight and improving cardiovascular risk factors in obese subjects [24], [25], [42]–[44]. However, studies of the obesity regulation effects of rimonabant mainly focus on the plasma/serum biochemical and lipid profile [45]–[47]. Less is known about the regulation of individual plasma and hepatic lipid species upon rimonabant treatment in obese subjects.

The present study describes the rearrangement and relocalisation of lipids in an early obese ApoE*3Leiden.CETP mouse model with humanized lipoprotein metabolism exposed to a 4-week rimonabant intervention. Lipoprotein profiling and lipidomics approaches were used for this purpose.

Throughout the experiment, food intake upon rimonabant dropped sharply during the first two days but quickly recovered to some extent during the first 3 weeks; since day 21, food intake has returned to nearly the levels in identical high-fat fed mice without rimonabant treatment. In spite of the recovery of energy intake within 3 weeks, the rimonabant group exhibited a significant and maintained weight reduction throughout the intervention period as compared with the non-treated controls. Collectively, this body weight reduction may be attributed to 1) the transient reduction in energy intake; 2) a reduction in energy efficiency (i.e. wastage of energy) caused by interruption of cannabinoid signalling upon rimonabant treatment [48]. Notably, the 9.4% body weight reduction induced by rimonabant treatment in our study seems not to be comparable with the at least 20% body weight loss induced by rimonabant reported in literature for obese mice or humans [42], [43]. However, it should not be neglected that the mouse model used in our study was in the early stage of obesity with only mildly increased body weight and plasma Cho.

Rimonabant intervention studies both in rodents and humans [24], [25], [46], [47], [49] have shown significant reduction of plasma TC and TG and a significant increase in HDL-C concentrations. Our 4-week rimonabant treatment of ApoE*3Leiden.CETP transgenic mice in early stage of obesity showed similar reduction of plasma TC and a reduction trend of plasma TG. However, the treatment did not significantly increase levels of HDL-C, which was probably due to our short intervention period combined with an early stage of obesity in our animal model. In line with these observations, plasma Cho and TG in VLDL were reduced, whereas Cho in HDL was slightly increased after the rimonabant intervention. These effects may be mediated via adiponectin, a major adipocyte cytokine involved in the regulation of hyperglycaemia, hyperinsulinaemia, and fatty acid oxidation at the peripheral adipocyte level [44], [50], [51]. Adiponectin release from adipocytes is known to be regulated by inhibition of CB_1_ [43], [52]. Furthermore, no significant reduction effect of rimonabant on CETP activity or levels was found in the ApoE*3Leiden mouse model.

The LC–MS-based plasma lipidomics analysis revealed a significant decrease of a large number of plasma lipids and a significant reduction in plasma PE, ChoE and TG lipid classes after rimonabant intervention, indicating the beneficial effect of rimonabant on plasma lipid metabolism in early stage of obesity. Specifically, as ChoE, TG and PLs (e.g. PE) are the main components of VLDL particles, their reduction in plasma indicated a lower circulated VLDL induced by rimonabant, which is in line with the result of plasma lipoprotein profiles, i.e., the VLDL fractions of Cho, TG and PLs were all reduced in rimonabant group. Evidence from animal studies and clinical trials indicated that the beneficial metabolic effects of rimonabant on plasma/serum lipid profiles are caused by the absence of basal endocannabinoid signaling, leading to reduced energy efficiency of food [24], [48], [53]. In addition, after multiple testing corrections six plasma lipid species showed a *most* significant reduction in response to rimonabant except for SPM (16∶0) which increased. Although it is unknown if these lipid compounds would be useful for understanding the pharmacological manipulation of rimonabant on improving obesity-related metabolic abnormalities under current experimental conditions, it may point to an important role of individual lipid molecular species in rimonabant's influence on the management of obesity.

It is recognized that high-fat diet-induced obesity is highly associated with a fatty liver due to the expression of the hepatic CB_1_ receptor [49], [54]. Liver-specific deletion of CB_1_ or blockage of the CB_1_ receptor was frequently used to protect against obesity-associated hepatic steatosis [47], [49], [53]. The results from available studies suggest that rimonabant, as a CB_1_ receptor antagonist, potentially has clinical applications in the treatment of high-fat diet-induced liver diseases [47], [53]. In the present study, down-regulation of lipids was not observed in liver tissues as it is in plasma after rimonabant treatment. This could be due to the early stage of obesity in our animal model, which displays a mild increase in body weight and moderately elevated plasma Cho levels (14–18 mmol/L). It also needs to be noted that our animal model is very different from those used in literature to investigate rimonabant's management in high-fat diet-induced liver disease. Our ApoE*3Leiden.CETP mice express a natural mutation of the human APOE3 gene in addition to the human APOC1 gene. Introduction of these genes induces an attenuated clearance of apoB-containing lipoproteins via the LDL receptor pathway [34], [55]. Mice with such a genetic background show mildly increased Cho and TG levels on a chow diet and a human-like lipoprotein profiles on high fat diet [56]. In summary, we proposed that 4-week rimonabant treatment has a moderate effect on liver lipid metabolism in the early stage of obesity of ApoE*3Leiden.CETP mice under current experimental conditions.

This study shows that LC–MS lipidomics approaches is promising for discovery of lipid biomarkers in relation to disease prevention and health promotion. Moreover, it was demonstrated that a 4-week rimonabant intervention improves body weight and cardiovascular risk factors during the early stage of obesity in ApoE*3Leiden.CETP mice. Finding of only limited amount of significant lipid changes may be attributed to the early stage of obesity in the animal model used. Taken together, it indicates that the effects of rimonabant on body weight and cardiovascular risk factors are moderate in the case of early stage obesity.

## Supporting Information

Text S1 General lipidomics protocol and liver lipidomics platform characteristics.
(DOC)

Figure S1 An example of a typical LC–MS chromatogram from a mouse liver total lipid extracts in ESI^+^ mode.
(TIF)

Figure S2 PCA of plasma and liver lipidomics data was applied to differentiate the nontreated controls (n = 8) and the animals treated with rimonabant (n = 8) of plasma and liver, respectively. PCA scores plot for all plasma samples (A) and all liver samples (B) from mean centred plus unit variance scaled data. Mouse marked with 3733 from the controls is an outlier of the PCA model for both the plasma lipidomics data and the liver lipidomics data.
(TIF)

Figure S3 Calibration curves for validation standard mixture (added to samples before lipid extraction). The calibration curves for each validation standard were determined from mouse liver total lipid extracts. Calibration curves of LPC (19∶0) at 0.5∼180 µg/ml (A) and 30∼180 µg/ml (B); of PC (38∶0) at 2.5∼450 µg/ml (C) and 150∼900 µg/ml (D); of PE (30∶0) at 1.5∼270 µg/ml (E) and 90∼540 µg/ml (F); and of TG (45∶0) at 1.5∼270 µg/ml (G) and 90∼540 µg/ml (H).
(TIF)

Table S1 General information of 8 exogenous lipid standards used in lipidomics analyses.
(DOC)

Table S2 The spiked concentrations of 8 exogenous lipid standards used in lipidomics analyses and normalization strategies used for LC-MS lipidomics data analyses.
(DOC)

Table S3 The RSD of the peak area ratios of lipids in study samples to corresponding lipid standards calculated in all QC samples.
(DOC)

Table S4 Experimental design for method validation of liver lipidomics profiling.
(DOC)

Table S5 Linearity (*R^2^*) for the four lipids from the validation mixture spiked to mouse liver samples prior to sample preparation.
(DOC)

Table S6 Intra-day and inter-day RSDs of the selected lipids in the sample (peak areas were normalized to those of corresponding IS).
(DOC)

Table S7 Variation in the retention time (denoted as mean ± S.D. min) of 8 exogenous lipid standards spiked with matrix at C_4_, C_6_ and C_8_ levels for repeatability during 3-day experiments.
(DOC)

Table S8 Recoveries of four lipids from the validation standard mixture at three (i.e. C_4_, C_6_ and C_8_) spiking concentrations.
(DOC)
