# Supplementary material for: Plasma and Liver Lipidomics Response to an Intervention of Rimonabant in ApoE*3Leiden.CETP Transgenic Mice
Source: PLoS One. 2011 May 17;6(5):e19423. doi: 10.1371/journal.pone.0019423 (PMC3096625; doi:10.1371/journal.pone.0019423)
Supplement: Text S1 — General lipidomics protocol and liver lipidomics platform characteristics. (DOC) [file pone.0019423.s001.doc]

Materials. Synthetic lipid standards including lyso-phosphocholine (LPC-17:0, LPC-19:0), phosphatidylethanolamine (PE-30:0, PE-34:0) and phosphatidylcholine (PC-34:0, PC-38:0) were purchased from Avanti Polar Lipids, Inc. (Alabaster, AL, USA) and triacylglyceride (TG-45:0, TG-51:0) from Sigma-Aldrich Chemie B.V. (Zwijndrecht, The Netherlands).

ULC-MS grade of acetonitrile (ACN), methanol (MeOH), isopropanol (IPA), and water as well as LC-MS grade of dichloromethane (CH_2_Cl_2_) were obtained from Biosolve (Valkenswaard, The Netherlands). Ammonium formate (AmFm, 99.995%) was of LC-MS grade and purchased from Sigma-Aldrich Chemie (St. Louis, MO, USA).

Rimonabant was purchased from Sanofi-Aventis Netherlands B.V. (Gouda, The Netherlands).

Lipid standards for plasma lipidomics profile and liver lipidomics profile. In brief, stock solutions of 8 lipid standards from 4 different lipid classes were separately prepared in a CH_2_Cl_2_ /MeOH mixture (2:1, v/v) for all standards except TG by weighing an exact amount of each lipid standard in new glass autosampler vials and stored at −20°C until further use. The TG standard was dissolved in CH_2_Cl_2_ instead of a CH_2_Cl_2_ /MeOH mixture due to its weak polarity.

For LC-MS lipidomics analysis of study samples, the stock solutions were separated into two sets. Set 1 consist of LPC (19:0), PC (38:0), PE (30:0) and TG (45:0), which was used for quantification of less intermediate → high abundant lipids in the samples; set 2 consist of LPC (17:0), PC (34:0), PE (34:0) and TG (51:0), which was used for quantification of very low → low abundant lipids in the samples. The final working solution for each set was prepared by pipetting a certain volume of corresponding stock solutions, “thawed” to room temperature followed by thorough vortex, into a new glass autosampler vial and diluting it to appropriate concentrations with a CH_2_Cl_2_ /MeOH mixture (2:1, v/v).

For method validation of LC-MS liver lipidomics profile, two sets of standard mixture were also used. Set 1 was used as internal standard (IS) mixture, consisting of LPC (1 7:0) at 30 μg/mL, PC (34:0) at 180 μg/mL, PE (34:0) at 120 μg/mL, and TG (51:0) at 90 μg/mL in a CH_2_Cl_2_ /MeOH mixture (2:1, v/v); and set 2 was used as “validation standard mixture” containing LPC (19:0), PC (38:0), PE (30:0), and TG (45:0) at the following C_0_−C_8_ concentration levels: LPC (19:0) at 0, 0.1, 0.5, 1, 2.5, 10, 30, 90, 180 μg/mL; PC (38:0) at 0, 0.5, 2.5, 5, 12.5, 50, 150, 450, 900 μg/mL; PE (30:0) and TG (45:0) at 0, 0.3, 1.5, 3, 7.5, 30, 90, 270, 540 μg/mL. From C_1_ to C_8_, the concentration was gradually increased, i.e., working solution of C_8_ was prepared first and then gradually diluted towards C_7_–C_1_. Three different concentrations (e.g. C_4_, C_6_ and C_8_, corresponding to low, medium and high concentration levels, respectively), were selected for evaluation of repeatability and calculation of recovery.

Liver samples for method validation of LC-MS liver lipidomics profile. Liver tissues from five healthy male mice at age of 8-12 weeks with C57BL/6 background were used for method validation of LC-MS liver lipidomics profile. These samples were kindly provided by Division of Toxicology, Leiden/Amsterdam Center for Drug Research, Leiden University (The Netherlands).

Liver sample pre-processing. The frozen liver samples stored at −80°C were immediately put into a lyophilizer after taken out from the freezer and lyophilized for 48 hours for the purpose of full dryness. After lyophilization, the liver was placed on clean aluminum foil followed by folding of the aluminum foil in order to cover the liver within it. A hammer was then used to triturate the crisp dry liver, wrapped inside the aluminum foil. Ten milligrams of liver powder was weighed in a clean 1.5 mL eppendorf vial for the subsequent lipid extraction. Notably, the triturated liver tissues from 5 mice used for method validation were mixed homogeneously before use.

Lipid extraction for method validation of liver lipid profiling. i) Spiking before sample preparation. Sixty microliters of IS mixture and 60 μL of the validation standard mixture were added to 10 mg of dry liver powder followed by addition of 160 μL of MeOH containing 0.02% antioxidant butylated hydroxytoluene (BHT), and then 320 μL of CH_2_Cl_2_ was added. The mixture was vortexed for 1 min both before and after CH_2_Cl_2_ addition. After that, the resulted suspension was placed for 5 min in an ultrasonic bath at −4°C and then placed in a shaker followed by 45 min incessantly shaking at −4°C. Then 10 min centrifugation at a rotation speed of 6000g at 10°C was needed before 500 μL of the supernatant was transferred into a new 1.5 ml eppendorf vial. 100 μL of 0.9% NaCl was subsequently added to the supernatant to give rise to a two-phase system: the nonlipid compounds were located in the upper aqueous phase, while most of the lipids were in the lower organic phase. After being centrifuged at 2000g at 10°C for 10 min, a total of 300 μL of lipid extract was collected from the bottom organic phase followed by addition of 60 μL of a CH_2_Cl_2_/MeOH mixture (2:1, v/v). Diluted the lipid extracts 40× with ACN/IPA/water (65:30:5, v/v/v); 10 μL was loaded for LC-MS lipidomics analysis.

ii) Spiking after sample preparation. The same procedures as described for spiking before sample preparation were conducted except that 60 μL of 2:1 CH_2_Cl_2_/MeOH instead of 60 μL of validation standard mixture was added to 10 mg dry liver powder before sample preparation, while 60 μL of the validation standard mixture instead of 60 μL 2:1 CH_2_Cl_2_/MeOH was added into the collected 300 μL of liver lipid extract.

QC samples. For the purpose of monitoring the LC-MS response in time and assessing data quality, quality control (QC) samples, were obtained by pooling all plasma or liver samples. Sample preparation for QC samples was conducted as the same manner as study samples of interest. The number of QC samples (number of aliquots of pooled plasma or pooled liver samples) needed depends on the size of the study. In the present study, two injections of one QC sample are performed after every 6^th^ study sample injections.

LC-MS chromatography. Lipid extracts were performed on a liquid chromatography-linear ion trap-Fourier transform ion cyclotron resonance-mass spectrometric (LC-FTMS) system equipped with a Surveyor HPLC MS pump, an autosampler (Thermo Fischer, San Jose, CA) and an Ascentis Express C8 2.1 × 150 mm (2.7 μm particle size) column (Sigma-Aldrich Chemie B.V., Zwijndrecht, The Netherlands). The binary gradient system consisted of water/ACN (2:3, 10 mM ammonium formate) and ACN/IPA (1:9, 10 mM ammonium formate). The performance of the elution for the gradient was identical to that reported before [1]. The flow rate was 0.26 mL/min. The column oven was maintained at 55°C and the temperature of the autosampler tray was 12°C.

The lipidomics profiling was carried out on Thermo Fischer linear ion trap-Fourier transform ion cyclotron resonance-mass spectrometry in the full ESI positive scan mode with a mass range of m/z 430-1500. IS of PC (34:0) was used to tune the mass spectrometry for optimizing the MS parameters. During tuning, 1 μg/mL of PC (34:0) at a flow rate of 10 μL/min from a Hamilton syringe pump and 30% A/70% B of elution at a flow rate of 250 μL/min from the Surveyor MS pump were continuously flowing into the LC-MS system. Finally, the optimized MS parameters are as following: the heated capillary was at 300°C; the voltages of the sampling cone and capillary were 3.8 kV and 48 V, respectively; the tube lens was 140 V. Nitrogen was used as sheath gas (60 units), auxiliary gas (5 units) and sweep gas (3 units). The LC−MS data were acquired by Xcalibur (Thermo Fisher) with 200 ms maximum injection time. The number of μscans was 2. Both the ion trap and FT scan events were recorded during data acquisition.

An example of a typical LC−MS chromatogram from a mouse liver lipid extract in ESI^+^ mode is shown in **Supplementary Figure S1**.

Data processing. Lipid peaks including spiked internal standards (IS) were extracted based on their expected retention time and accurate masses according to an in-house lipid database using LCquan v2.5 (Thermo Fisher). The extracted lipid peaks were initially integrated by LCquan and manually corrected if needed. The parameters for the integration were peak detection algorithm, ICIS; smoothing points, 7; window, 60 s; view width, 3 min; baseline, 100; area noise factor, 5; peak noise factor, 10. The LCquan pre-processed data were exported as an Excel report. The standards, used for quantification, were picked out from the pre-processed Excel data sheet and the relative standard deviation (RSD) of their absolute peak areas from all injected samples was subsequently calculated to check the stability of the LC−MS system during the measurement. Lipids detected in study samples were quantified as the peak area ratios of lipids in study samples to lipid standards. After quantification, duplicate measurements were combined into a single measurement. And then data quality was assessed by calculating the RSD of the peak area ratios of lipids in study samples to appropriate lipid standards in all QC samples. Peaks with a %RSD more than 20% were excluded leaving 131 lipids in plasma lipidomics data set and 133 lipids in liver lipidomics data set for further data analysis.

The lipidomics datasets were first analyzed by independent student t-test and late extended with Benjamini and Hochberg multiple testing corrections. *P*<0.05 was considered to be statistically significant. In order to visualize possible relations between the samples from treated and non-treated groups, principal component analysis (PCA) was carried out for the mean centered plus unit variance scaled plasma lipidomics data set and liver lipidomics data set, respectively using Matlab software (version 6.5.1, release 13, The Mathworks, 2003).

One control mouse (marked as 3733) was excluded from statistical data analyses throughout the article, because it did not respond to Western-type diet during run-in period and failed to reach hypercholesterolemia criteria essential for our experiment. **Supplementary Figure S2** shows the PCA scores plot of all plasma (A) and liver samples (B) in which mouse 3733 is an outlier in both cases.

Analytical characteristics of liver lipidomics profiling. In order to ensure an adequate performance in metabolomics studies of lipids in tissue based matrix, the analytical characteristics in terms of linearity, limit of detection (LOD), repeatability and recovery were selected for validation as is proposed by ICH [2] and IUPAC guidelines [3]. Experimental design for method validation of liver lipidomics profiling is shown in **Supplementary Table S4.**

**Linearity.** Four exogenous lipid standards from “validation standard mixture” were used to determine the linearity of lipids in liver tissue. Concentrations at 9 different levels of the standards including a blank sample (C_0_, matrix sample processed without standards) and eight non-zero samples (C_1_-C_8_, matrix sample processed with standards) were chosen on the basis of the concentration range expected in lipidomics study of liver tissues. The experiment was performed by spiking the liver matrix with known concentration of the validation standards and four IS before sample preparation. At each concentration level, the peak areas of either four or six replicate measurements were calculated. For each validation standard, the calibration curve was constructed using the mean of the peak area ratios of this standard to the corresponding IS. The calibration curves were evaluated by calculating the linear regression coefficient of determination (R^2^) and the RSD (see **Supplementary Table S5**). Different compounds showed different linear ranges: 0.5−180 μg/mL was for LPC (19:0), 0.5−270 μg/mL was for PE (30:0) and TG (45:0), and 1.5−450 μg/mL was for PC (38:0) (see **Supplementary** **Figure S3A-H** for the calibration curves for lipid standards at different concentration ranges).

**LOD.** LOD determined on the basis of response at a signal-to-noise ratio (S/N) of 3 was 10 pg for LPC (19:0), 15 pg for PE (30:0), 1.5 pg for PC (38:0) and 0.5 pg for TG (45:0).

**Repeatability.** The repeatability of the analytical platform was assessed by repeatedly performing the procedures of sample preparation on multiple aliquots of pooled mouse liver powder from 5 male mice and analyzing the extracted samples on three consecutive days. Three levels of concentrations covering the range of expected concentrations were chosen for the repeatability estimates as proposed by IUPAC [3]. Briefly, at each spiking concentration three aliquots of pooled mouse liver were extracted spiked with the validation standard mixture together with IS mixture prior to sample preparation; each lipid extraction was injected in duplicate (n = 3 × 2). To estimate the repeatability, fifteen lipid molecular species from five different lipid classes were selected on the basis of the peak intensity (e.g., low, medium and high abundance) and the retention time window. **Supplementary Table S6** summarized the Intra-day and inter-day RSDs of the selected lipids obtained at low, medium and high spiking concentrations, respectively. The repeatability determined with a RSD less than 15% was sufficiently satisfactory for lipidomics research at the level of tissues. Meanwhile, the variation in the retention time (denoted as mean ± S.D.) of eight lipid standards during 3-day experiment were presented in **Supplementary Table S7**.

**Recovery.** The recovery experiment was conducted for four lipids from the validation standard mixture, which were spiked before and after extraction at low (C_4_), medium (C_6_) and high (C_8_) spiking levels, respectively (see section of *lipid standards for plasma lipidomics profile and liver lipidomics profile* in **Supplementary** text). Notably, a mixture of four IS was invariably spiked prior to extraction in both of above mentioned procedures for the purpose of intensity correction [4]. Three aliquots were prepared for each spiked level of validation standard mixture and each was injected in duplicates. In total, 36 aliquots of pooled mouse liver were used for the recovery experiment, i.e. 18 aliquots (3 + 3 aliquots for each of the three spiking concentration levels) were extracted on day 2; 18 aliquots (3 + 3 aliquots at medium spiking concentration level for each of the three days) were extracted on 3 consecutive days (See experimental design for method validation of liver lipidomics profiling summarized in **Supplementary Table S4** for details). The recoveries for each of the validation standard were calculated as the mean ratio of IS normalized peak area ratio of that lipid standard spiked before extraction to that lipid standard spiked after extraction, and multiplied by a coefficient of 1.45 called R_coeff_. and then by 100. The R_coeff_. was obtained according to the following formula:

R_coeff_. = [(500/600) × (290/350)]^-1^

In the formula, 500 represents 500 μL of crude lipid extract transferred, and 600 represents the total volume of the resulted suspension (e.g. total crude lipid extract) before addition of 100 μL of 0.9% NaCl. 290 represents 290 μL of lipid extract transferred from the bottom organic phase after addition of 0.9% NaCl, and 350 represents the total volume of the bottom organic phase (See section of lipid extraction for liver samples in main text of the article).

The R_coeff_. is used to calculate the loss of the standards spiked before sample preparation back. It specially needs to be considered in our experimental set up to avoid obtaining biased results, since the concentration of validation standard mixture for spiking is the same in both spiking before and spiking after situations. Under the situation of spiking before, only partial lipid extracts can be transferred during 2 times of extract collection, e.g. only 500 out of 600 μL crude lipid extracts and only 290 out of 350 μL lipid extracts were transferred during the first and the second transferring procedures, respectively. That means almost 31% of original exogenous lipids spiked before extraction is lost during the experiment.

The recoveries for four exogenous lipids at three spiked concentrations are displayed in **Supplementary Table S8**. The recoveries obtained at both low (C_4_) and medium (C_6_) spiked levels for four lipid standards were comparable. Relatively higher recoveries for PE (30:0), PC (38:0) and TG (45:0) at high spiked level (C_8_) were obtained as compared to those spiked at low and medium level.

**Supplementary References**

1. Hu CX, van Dommelen J, van der Heijden R, Spijksma G, Reijmers TH, et al. (2008) RPLC-Ion-Trap-FTMS Method for Lipid Profiling of Plasma: Method Validation and Application to p53 Mutant Mouse Model. J Proteome Res 7: 4982-4991.

2. ICH HARMONISED GUIDELINE on ‘VALIDATION OF ANALYTICAL PROCEDURES: TEXT AND METHODOLOGY’ Q2 (R1); Current Step 4 version; Parent Guideline dated 27 October 1994; (Complementary Guideline on Methodology dated 6 November 1996 incorporated in November 2005); established by International Conference on Harmonisation of Technical Requirements for Registration of Pharmaceuticals for Human Use.

3. Thompson M, Ellison SLR, and Wood R (2002) Harmonized guidelines for single-laboratory validation of methods of analysis - (IUPAC technical report). Pure and Applied Chemistry 74: 835-855.

4. Bijlsma S, Bobeldijk I, Verheij ER, Ramaker R, Kochhar S, et al. (2006) Large-scale human metabolomics studies: a strategy for data (pre-) processing and validation. Anal Chem 78: 567-574.
