# Supplementary figures and images for: Plasma and Liver Lipidomics Response to an Intervention of Rimonabant in ApoE*3Leiden.CETP Transgenic Mice
Source: PLoS One. 2011 May 17;6(5):e19423. doi: 10.1371/journal.pone.0019423 (PMC3096625; doi:10.1371/journal.pone.0019423)

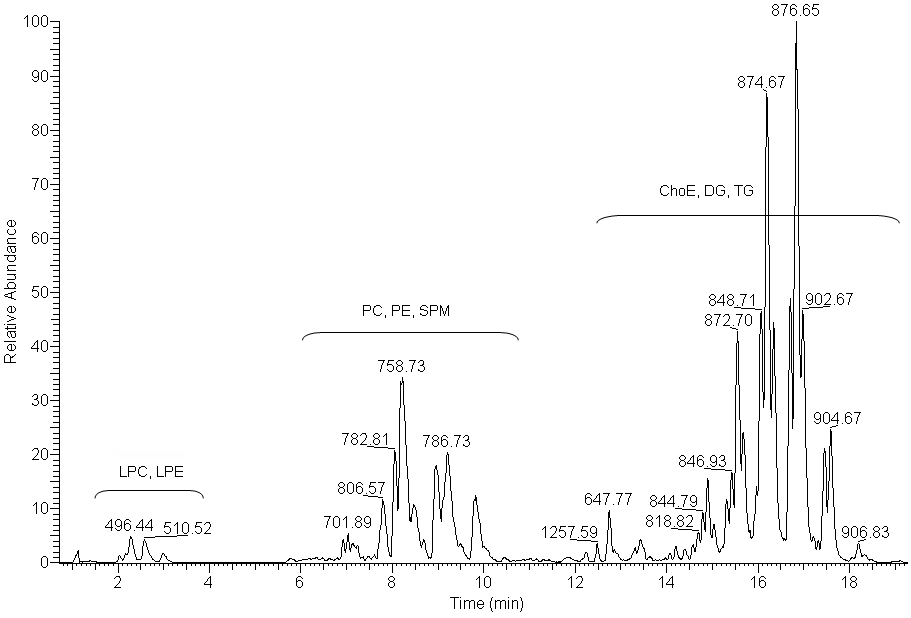

Supplement: Figure S1 — An example of a typical LC–MS chromatogram from a mouse liver total lipid extracts in ESI+ mode. (TIF) [file pone.0019423.s002.tif]

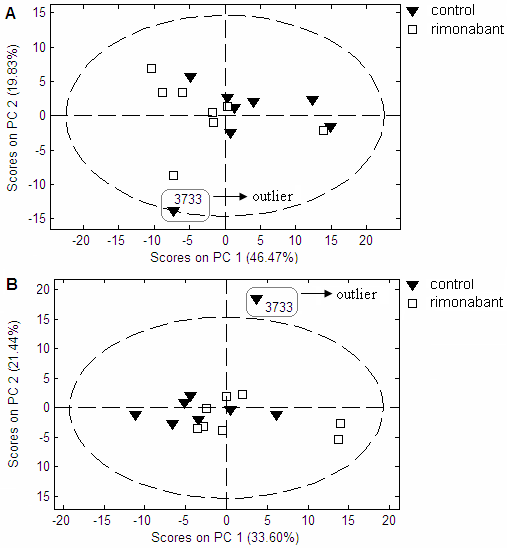

Supplement: Figure S2 — PCA of plasma and liver lipidomics data was applied to differentiate the nontreated controls (n = 8) and the animals treated with rimonabant (n = 8) of plasma and liver, respectively. PCA scores plot for all plasma samples (A) and all liver samples (B) from mean centred plus unit variance scaled data. Mouse marked with 3733 from the controls is an outlier of the PCA model for both the plasma lipidomics data and the liver lipidomics data. (TIF) [file pone.0019423.s003.tif]

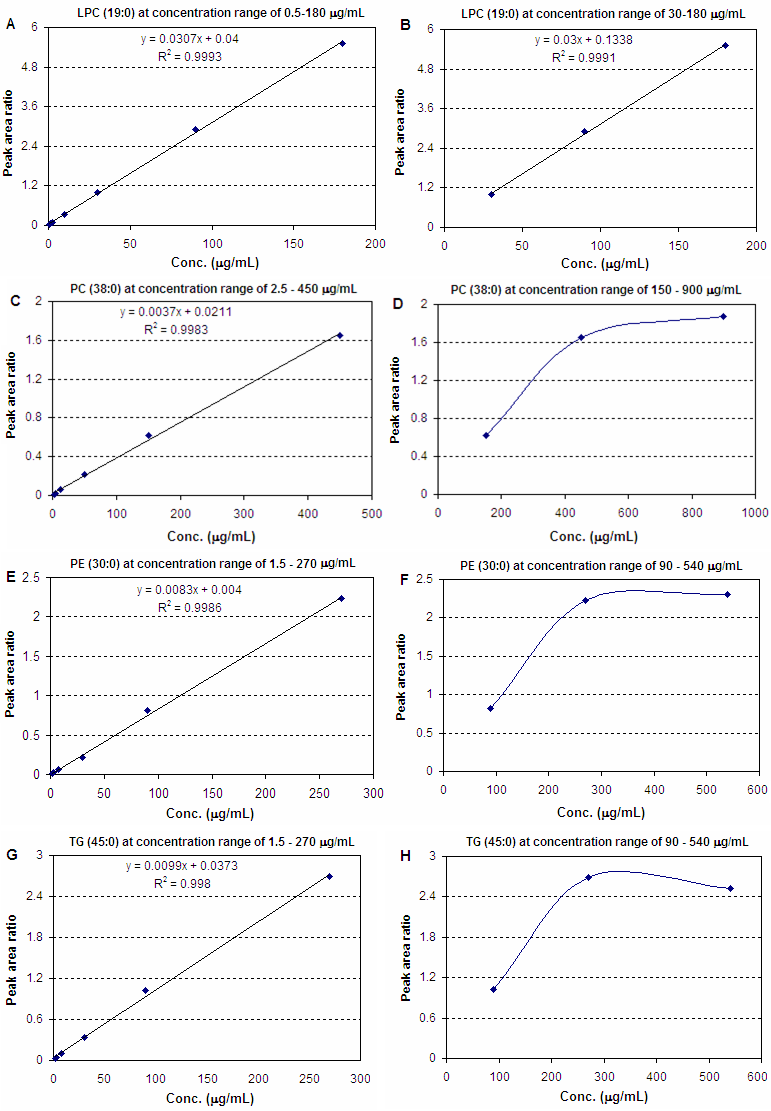

Supplement: Figure S3 — Calibration curves for validation standard mixture (added to samples before lipid extraction). The calibration curves for each validation standard were determined from mouse liver total lipid extracts. Calibration curves of LPC (19∶0) at 0.5∼180 µg/ml (A) and 30∼180 µg/ml (B); of PC (38∶0) at 2.5∼450 µg/ml (C) and 150∼900 µg/ml (D); of PE (30∶0) at 1.5∼270 µg/ml (E) and 90∼540 µg/ml (F); and of TG (45∶0) at 1.5∼270 µg/ml (G) and 90∼540 µg/ml (H). (TIF) [file pone.0019423.s004.tif]
