# Supplementary material for: Plasma and Liver Lipidomics Response to an Intervention of Rimonabant in ApoE*3Leiden.CETP Transgenic Mice
Source: PLoS One. 2011 May 17;6(5):e19423. doi: 10.1371/journal.pone.0019423 (PMC3096625; doi:10.1371/journal.pone.0019423)
Supplement: Table S1 — General information of 8 exogenous lipid standards used in lipidomics analyses. (DOC) [file pone.0019423.s005.doc]

##### Table S1. General information of 8 exogenous lipid standards used in lipidomics analyses.

| Lipid | Monoisotopic |  | Plasma lipidomics | | | Liver lipidomics | | |
| --- | --- | --- | --- | --- | --- | --- | --- | --- |
| standard | mass | Ion adduct | stock | working | stock | | working |  |
|  | (m/z) |  | (mg/ml) | (µg/ml) | (mg/ml) | | (µg/ml) |  |
| LPC (17:0) | 510.3557 | [M + H]^+^ | 0.75 | 6 | 0.75 | | 6 |  |
| PE (34:0) | 720.5583 | [M + H]^+^ | 1.05 | 20 | 1.80 | | 30 |  |
| PC (34:0) | 762.6025 | [M + H]^+^ | 1.20 | 20 | 1.50 | | 50 |  |
| TG (51:0) | 866.8209 | [M + NH_4_]^+^ | 1.25 | 20 | 2.25 | | 180 |  |
| LPC (19:0) | 538.3880 | [M + H]^+^ | 0.90 | 120 | 0.75 | | 72 |  |
| PE (30:0) | 664.4927 | [M + H]^+^ | 2.50 | 120 | 2.45 | | 360 |  |
| PC (38:0) | 818.6658 | [M + H]^+^ | 3.50 | 600 | 3.75 | | 600 |  |
| TG (45:0) | 782.7264 | [M + NH_4_]^+^ | 2.50 | 240 | 4.20 | | 1920 |  |
