# Supplementary material for: Plasma and Liver Lipidomics Response to an Intervention of Rimonabant in ApoE*3Leiden.CETP Transgenic Mice
Source: PLoS One. 2011 May 17;6(5):e19423. doi: 10.1371/journal.pone.0019423 (PMC3096625; doi:10.1371/journal.pone.0019423)
Supplement: Table S2 — The spiked concentrations of 8 exogenous lipid standards used in lipidomics analyses and normalization strategies used for LC-MS lipidomics data analyses. (DOC) [file pone.0019423.s006.doc]

**Table S2. The spiked concentrations of 8 exogenous lipid standards used in lipidomics analyses and normalization strategies used for LC-MS lipidomics data analyses.**

| Lipid | Spiked conc. (µg/mL) | | Quantified | Quantified lipids' | %RSD | |
| --- | --- | --- | --- | --- | --- | --- |
| standards | plasma | liver | lipids | abundance | plasma | liver |
| LPC (17:0) | 1.5 | 1.5 | LPC, LPE | very low → low | 13.6 | 14.7 |
| PE (34:0) | 5 | 7.5 | PE |  | 23.9 | 26.3 |
| PC (34:0) | 5 | 12.5 | PC, SM |  | 9.0 | 14.8 |
| TG (51:0) | 5 | 45 | ChoE, TG |  | 12.2 | 13.0 |
| LPC (19:0) | 30 | 18 | LPC | less intermediate | 8.3 | 14.4 |
| PE (30:0) | 30 | 90 | PE | → high | 9.3 | 16.0 |
| PC (38:0) | 150 | 150 | PC, SM |  | 10.8 | 13.6 |
| TG (45:0) | 60 | 480 | TG |  | 11.0 | 11.4 |
