# Supplementary material for: Plasma and Liver Lipidomics Response to an Intervention of Rimonabant in ApoE*3Leiden.CETP Transgenic Mice
Source: PLoS One. 2011 May 17;6(5):e19423. doi: 10.1371/journal.pone.0019423 (PMC3096625; doi:10.1371/journal.pone.0019423)
Supplement: Table S3 — The RSD of the peak area ratios of lipids in study samples to corresponding lipid standards calculated in all QC samples. (DOC) [file pone.0019423.s007.doc]

**Table S3. The RSD of the peak area ratios of lipids in study samples to corresponding lipid standards calculated** in all QC samples

| Plasma lipidomics dataset | | Liver lipidomics dataset | |
| --- | --- | --- | --- |
| %RSD | Number of peaks | %RSD | Number of peaks |
| 0 - 5 | 45 | 0 - 5 | 71 |
| 5 - 10 | 56 | 5 - 10 | 47 |
| 10 - 15 | 19 | 10 - 15 | 8 |
| 15 - 20 | 11 | 15 - 20 | 7 |
| > 20 | 1 | > 20 | 3 |
