# Supplementary material for: Plasma and Liver Lipidomics Response to an Intervention of Rimonabant in ApoE*3Leiden.CETP Transgenic Mice
Source: PLoS One. 2011 May 17;6(5):e19423. doi: 10.1371/journal.pone.0019423 (PMC3096625; doi:10.1371/journal.pone.0019423)
Supplement: Table S4 — Experimental design for method validation of liver lipidomics profiling. (DOC) [file pone.0019423.s008.doc]

**Table S4. Experimental design for method validation of liver lipidomics profiling.**

| Sample characteristics used for  validation experiments | | | Validation parameters investigated | | | | | |  |
| --- | --- | --- | --- | --- | --- | --- | --- | --- | --- |
| Concentration | Biol. sample | Added prior sample prep. | Calibration line | Repeatability | Intermediate precision | Recovery, extraction efficiency, ion suppression | | LLOQ | Total number ^e^ |
|  |  |  | Number of samples on days 1-3 | | | | | |  |
|  |  |  | Day1 | Day1 | Day 2&3 | Day 1-3 | Day 2 |  |  |
| C0 | x | No | 2 |  |  |  |  |  | 4 |
| C1 | x | Very low | 2 |  |  |  |  | b | 4 |
| C2 | x | Less lower | 2 |  |  |  |  |  | 4 |
| C3 | x | Lower | 2 |  |  |  |  |  | 4 |
| C4 | x | Low | 2 ^a^ | 3 | 3 |  | 3 (IS) before + 3 after  Sample preparation ^c^ |  | 24 |
| C5 | x | Less intermediate | 2 |  |  |  |  |  | 4 |
| C6 | x | Intermediate | 2 ^a^ | 3 | 3 | 3 (IS) before + 3 after  Sample preparation ^d^ |  |  | 24 |
| C7 | x | Higher | 2 |  |  |  |  |  | 4 |
| C8 | x | High | 2 ^a^ | 3 | 3 |  | 3 (IS) before + 3 after  Sample preparation ^c^ |  | 24 |
| Total |  |  |  |  |  |  |  |  | 96 |

^a^ Three samples were virtually analyzed so that the data were shared for calibration line, intermediate precision and recovery determination.

^b^ Calculated from RSD of lowest concentration point of calibration line.

^c^ One sample matrix at three concentration (low, medium, and high) levels was investigated within one day.

^d^ One sample matrix at medium concentration was investigated on three consecutive days.

^e^ Number of injections
