# Supplementary material for: Plasma and Liver Lipidomics Response to an Intervention of Rimonabant in ApoE*3Leiden.CETP Transgenic Mice
Source: PLoS One. 2011 May 17;6(5):e19423. doi: 10.1371/journal.pone.0019423 (PMC3096625; doi:10.1371/journal.pone.0019423)
Supplement: Table S5 — Linearity (R2) for the four lipids from the validation mixture spiked to mouse liver samples prior to sample preparation. (DOC) [file pone.0019423.s009.doc]

**Table S5. Linearity (*R^2^*) *^a^* for the four lipids from the validation mixture spiked to mouse liver samples prior to sample preparation.**

|  |  | LPC (19:0)/LPC (17:0) | | | PE (30:0)/PE (34:0) | | | PC (38:0)/PC (34:0) | | | TG (45:0)/TG (51:0) | | |
| --- | --- | --- | --- | --- | --- | --- | --- | --- | --- | --- | --- | --- | --- |
| Conc. level*^b^* | n | conc.*^c^* | mean | RSD | conc. | mean | RSD | conc. | mean | RSD | conc. | mean | RSD |
|  |  | (µg/ml) | peak area | (%) | (µg/ml) | peak area | (%) | (µg/ml) | peak area | (%) | (µg/ml) | peak area | (%) |
|  |  |  | ratio*^d^* |  |  | ratio |  |  | ratio |  |  | ratio |  |
| C_0_ | 4 | 0 | 0 |  | 0 | 0 |  | 0 | 0 |  | 0 | 0 |  |
| C_1_ | 4 | 0.1 | 0.014 | 1.3 | 0.3 | 0 | 0 | 0.5 | 0.004 | 1 | 0.3 | 0.006 | 2.4 |
| C_2_ | 4 | 0.5 | 0.028 | 4.8 | 1.5 | 0.01 | 18.4 | 2.5 | 0.011 | 3.8 | 1.5 | 0.023 | 5.6 |
| C_3_ | 4 | 1 | 0.048 | 2.2 | 3 | 0.02 | 8.9 | 5 | 0.023 | 1.9 | 3 | 0.045 | 4.5 |
| C_4_ | 6 | 2.5 | 0.098 | 2 | 7.5 | 0.067 | 8.1 | 12.5 | 0.057 | 1.2 | 7.5 | 0.105 | 1.5 |
| C_5_ | 4 | 10 | 0.336 | 2.3 | 30 | 0.222 | 15.6 | 50 | 0.219 | 2.1 | 30 | 0.33 | 10.4 |
| C_6_ | 6 | 30 | 0.987 | 2.3 | 90 | 0.814 | 14 | 150 | 0.62 | 3.9 | 90 | 1.025 | 13 |
| C_7_ | 4 | 90 | 2.913 | 1.3 | 270 | 2.23 | 4.6 | 450 | 1.654 | 2.5 | 270 | 2.689 | 4 |
| C_8_ | 6 | 180 | 5.565 | 3.1 | 540 | 2.459 | 21.7 | 900 | 1.945 | 1 | 540 | 2.623 | 2.4 |
| *R^2^* |  | 0.9993 | | | 0.9986 | | | 0.9983 | | | 0.998 | | |
| Linear range |  | 0.5 – 180 μg/ mL | | | 1.5 – 270 μg/ mL | | | 2.5 – 450 μg/ mL | | | 1.5 – 270 μg/ mL | | |

*^a^* Calculated from the equation *y* = *a*x + *b;* the calibration line was constructed using the mean ratios of the peak area of each lipid of the validation mixture to the peak area of the corresponding IS (both were spiked before sample extraction); *b* duplicate sample preparations at C_0_, C_1_, C_2_, C_3_, C_5_ and C_7_ while triplicate sample preparations at C_4_, C_6_ and C_8_ were performed and duplicate analyses of each extract were performed; *^c^* concentration of each lipid of the validation mixture spiked to liver sample; *^d^* the mean peak area ratios of the replicates.
