# Supplementary material for: Plasma and Liver Lipidomics Response to an Intervention of Rimonabant in ApoE*3Leiden.CETP Transgenic Mice
Source: PLoS One. 2011 May 17;6(5):e19423. doi: 10.1371/journal.pone.0019423 (PMC3096625; doi:10.1371/journal.pone.0019423)
Supplement: Table S6 — Intra-day and inter-day RSDs of the selected lipids in the sample (peak areas were normalized to those of corresponding IS). (DOC) [file pone.0019423.s010.doc]

**Table S6. Intra-day and inter-day RSDs of the selected lipids in the sample (peak areas were normalized to those of corresponding IS).**

| Selected | Low spiking | | Medium spiking | | High spiking | |
| --- | --- | --- | --- | --- | --- | --- |
| Lipids | Intra-day | Inter-day | Intra-day | Inter-day | Intra-day | Inter-day |
|  | (%RSD) | (%RSD) | (%RSD) | (%RSD) | (%RSD) | (%RSD) |
| LPC (16:0) | 2.99 | 3.56 | 3.13 | 3.32 | 2.39 | 2.7 |
| LPC (18:2) | 4.19 | 4.39 | 2.52 | 6.46 | 2.49 | 6.0 |
| LPC (22:6) | 4.21 | 4.21 | 2.64 | 6.53 | 2.84 | 5.45 |
| SPM (16:0) | 5.0 | 5.0 | 6.4 | 10.38 | 3.19 | 8.43 |
| SPM (20:0) | 4.22 | 9.95 | 7.42 | 11.0 | 5.33 | 5.39 |
| SPM (22:0) | 3.04 | 3.71 | 3.48 | 3.68 | 2.64 | 3.89 |
| PC (32:0) | 3.29 | 3.49 | 1.77 | 4.71 | 3.01 | 5.0 |
| PC (38:6) | 3.91 | 3.91 | 6.26 | 11.99 | 1.55 | 8.74 |
| PC (40:8) | 6.45 | 9.38 | 7.9 | 9.27 | 9.13 | 11.71 |
| PE (34:2) | 6.08 | 7.34 | 4.87 | 9.48 | 3.88 | 6.07 |
| PE (38:6) | 4.99 | 5.24 | 9.68 | 12.61 | 3.28 | 9.08 |
| PE (40:6) | 4.56 | 4.57 | 8.85 | 11.47 | 2.63 | 10.26 |
| TG (50:4) | 4.68 | 13.58 | 5.47 | 12.68 | 10.53 | 11.47 |
| TG (54:5) | 5.29 | 10.89 | 4.64 | 13.87 | 6.51 | 6.51 |
| TG (58:10) | 7.14 | 12.22 | 3.1 | 6.5 | 6.83 | 8.96 |
