# Supplementary material for: Plasma and Liver Lipidomics Response to an Intervention of Rimonabant in ApoE*3Leiden.CETP Transgenic Mice
Source: PLoS One. 2011 May 17;6(5):e19423. doi: 10.1371/journal.pone.0019423 (PMC3096625; doi:10.1371/journal.pone.0019423)
Supplement: Table S7 — Variation in the retention time (denoted as mean ± S.D. min) of 8 exogenous lipid standards spiked with matrix at C4, C6 and C8 levels for repeatability during 3-day experiments. (DOC) [file pone.0019423.s011.doc]

**Table S7. Variation in the retention time (denoted as mean ± S.D. min) of 8 lipid standards spiked with matrix at C_4_, C_6_ and C_8_ levels for repeatability during 3-day experiments.**

| Lipid | Observed | Day1 (n = 18) | Day2 (n = 18) | Day3 (n = 18) |
| --- | --- | --- | --- | --- |
| standards | mass (m/z) | Retention time (mean ± S.D., min) | | |
| LPC (17:0) | 510.34 | 2.56 ± 0.02 | 2.58 ± 0.003 | 2.59 ± 0.002 |
| LPC (19:0) | 538.35 | 3.46 ± 0.02 | 3.48 ± 0.004 | 3.50 ± 0.01 |
| PE (30:0) | 664.49 | 8.16 ± 0.03 | 8.19 ± 0.01 | 8.19 ± 0.01 |
| PE (34:0) | 720.55 | 10.02 ± 0.03 | 10.05 ± 0.01 | 10.05 ± 0.01 |
| PC (34:0) | 762.60 | 9.79 ± 0.03 | 9.85 ± 0.01 | 9.87 ± 0.01 |
| PC (38:0) | 818.66 | 11.65 ± 0.03 | 11.73 ± 0.02 | 11.77 ± 0.02 |
| TG (45:0) | 782.55 | 16.62 ± 0.03 | 16.63 ± 0.02 | 16.63 ± 0.02 |
| TG (51:0) | 866.66 | 19.23 ± 0.03 | 19.26 ± 0.02 | 19.24 ± 0.02 |
