# Supplementary material for: Plasma and Liver Lipidomics Response to an Intervention of Rimonabant in ApoE*3Leiden.CETP Transgenic Mice
Source: PLoS One. 2011 May 17;6(5):e19423. doi: 10.1371/journal.pone.0019423 (PMC3096625; doi:10.1371/journal.pone.0019423)
Supplement: Table S8 — Recoveries of four lipids from the validation standard mixture at three (i.e. C4, C6 and C8) spiking concentrations. (DOC) [file pone.0019423.s012.doc]

**Table S8. Recoveries of four lipids from the validation standard mixture at three (i.e. C_4_, C_6_ and C_8_) spiking concentrations.**

| Validation | Recovery (%, mean ± SD) | | |
| --- | --- | --- | --- |
| standards | low spiked (C_4_) | medium spiked (C_6_) | high spiked (C_8_) |
| LPC (19:0) | 80.6 ± 2.9 | 70.2 ± 3.5 | 75.1 ± 1.3 |
| PE (30:0) | 84.1 ± 3.9 | 91.3 ± 11.4 | 98.9 ± 4.5 |
| PC (38:0) | 86.7 ± 3.6 | 90.3 ± 5.3 | 111.1 ± 1.9 |
| TG (45:0) | 81.6 ± 5.4 | 93.1 ± 7.7 | 116.6 ± 5.4 |
